# Change in Evaluation Mode Can Cause a Cheerleader Effect

**DOI:** 10.3389/fpsyg.2021.607448

**Published:** 2021-04-21

**Authors:** Claude Messner, Mattia Carnelli, Patrick Stefan Höhener

**Affiliations:** Consumer Behavior, University of Bern, Bern, Switzerland

**Keywords:** cheerleader effect, change in evaluation mode, evaluation mode, hierarchical encoding, attractiveness

## Abstract

The *cheerleader effect* describes the phenomenon whereby faces are perceived as being more attractive when flanked by other faces than when they are perceived in isolation. At least four theories predict the cheerleader effect. Two visual memory processes could cause a cheerleader effect. First, visual information will sometimes be averaged in the visual memory: the averaging of faces could increase the perceived attractiveness of all the faces flanked by other faces. Second, information will often be combined into a higher-order concept. This hierarchical encoding suggests that information processing causes faces to appear more attractive when flanked by highly attractive faces. Two further explanations posit that comparison processes cause the cheerleader effect. While *contrast effects* predict that a difference between the target face and the flanking faces causes the cheerleader effect due to comparison processes, a *change in the evaluation mode*, which alters the standard of comparison between joint and separate evaluation of faces, could be sufficient for producing a cheerleader effect. This leads to the prediction that even when there is no contrast between the attractiveness of the target face and the flanking faces, a cheerleader effect could occur. The results of one experiment support this prediction. The findings of this study have practical implications, such as for individuals who post selfies on social media. An individual’s face will appear more attractive in a selfie taken with people of low attractiveness than in a selfie without other people, even when all the faces have equally low levels of attractiveness.

## Introduction

Barney Stinson, a main character in the television series *How I Met Your Mother*, hypothesized that people are perceived as being more attractive in a group than when perceived individually; he called this phenomenon the *cheerleader effect*. Walker and Vul (2014) introduced this term into scientific discourse and provided the first empirical evidence for the effect.

The cheerleader effect has been replicated several times (Carragher et al., 2018, 2019; Ying et al., 2019), and several human information processes predict this effect. While initial work assumes that visual memory processes could cause the cheerleader effect (Walker and Vul, 2014), recent research adds that the cognitive processes of judgment and decision-making could also cause cheerleader effects (Ying et al., 2019).

The aim of this article is to introduce a further cognitive process that could cause a cheerleader effect. Evaluation of an individual face can only be based on a comparison with the viewer’s internal standard, while evaluation of a face in a group can be based on contrast with other faces. This *change in the evaluation mode* can cause a cheerleader effect even if there is no contrast between the attractiveness of the target face and the flanking faces. If a face of low attractiveness is compared to an internal standard, it appears less attractive than when it is compared to other faces of low attractiveness. Therefore, even if there is no contrast between the attractiveness of the target face and the flanking faces, a cheerleader effect could emerge.

Evidence that a change in evaluation mode could cause a cheerleader effect does not mean that the other processes are false. Rather, in specific situations, the evaluation mode could predict a cheerleader effect that could not be explained by one of the other processes. At the end of this article, we discuss situations which could foster one of the other processes to cause cheerleader effects. The aim of this article is not to falsify alternative processes, but to provide initial evidence that a change in evaluation mode could cause a cheerleader effect.

We chose a design in which four theories differ in how they predict the cheerleader effect, and we chose a situation that corresponds to a real-time situation, one where an observer sees an unknown person for the first time. This has some relevance, for example, for people who decide to post a selfie on social media. The question is whether faces are perceived as more attractive in isolation or together with flanking faces. Furthermore, we want to investigate if it matters if the flanking faces are less, equally, or more attractive than the target face.

### Cheerleader Effect

The attractiveness of a face is influenced not only by its facial features (Fink and Penton-Voak, 2002; Said and Todorov, 2011), but also by other faces seen previously (Ying et al., 2019) or simultaneously (Walker and Vul, 2014; Carragher et al., 2018, 2019). The latter produces what is called the cheerleader effect. While some studies have failed to replicate the cheerleader effect (van Osch et al., 2015), others have successfully reproduced it (Carragher et al., 2018, 2019; Ying et al., 2019).

Some boundary conditions of the cheerleader effect have been tested in recent studies. The cheerleader effect occurs in natural group settings (Walker and Vul, 2014), in single pictures of faces (Walker and Vul, 2014), and with computer-generated faces (Ying et al., 2019). Neither the size of the group (Walker and Vul, 2014) nor the position of the target face (Carragher et al., 2018) moderate the cheerleader effect; however, the attractiveness of the flanking faces plays a moderating role: the less attractive the flanking faces, the greater the cheerleader effect (Ying et al., 2019).

Prior research suggests that visual memory processes could cause the cheerleader effect (Walker and Vul, 2014). Langlois and Roggman (1990) argue that *averaging* visual information could cause a cheerleader effect, because averaging increases the attractiveness of faces. This would predict that any target faces would be evaluated as more attractive when flanked by other faces than in isolation. Another visual memory process is *hierarchical encoding*, which could also predict a cheerleader effect, but only when faces are flanked by more attractive faces. Hierarchical encoding describes the process whereby information in the visual working memory is not stored independently but is constructed into a higher-order representation (Vogel et al., 2001; Luck and Vogel, 2013; Im and Chong, 2014; Oriet and Hozempa, 2016). Observers could encode a group of attractive faces as an attractive group. Less attractive faces within this group would profit from this hierarchical encoding and be perceived as more attractive. As a result, hierarchical encoding leads to a bias toward the mean. If an individual is shown an image containing several blue and red circles, wherein the blue circles are mostly large and the red circles mostly small, then a medium-size circle is remembered as being larger when it is blue than when it is red (Brady and Alvarez, 2011). In other words, the recall of individual items is biased toward the group mean, so faces are perceived as being more attractive when flanked by other more attractive faces than when perceived in isolation. Less attractive target faces may be expected to profit more from highly attractive flanking faces than highly attractive target faces would. When faces are flanked by faces of low attractiveness, hierarchical encoding would lead one to expect a reverse cheerleader effect: faces will be perceived as more attractive in isolation than when accompanied by other faces. If the attractiveness of the target face is equal to the mean of the flanking faces, hierarchical encoding would predict no effect.

Ying et al. (2019) introduced a cognitive aspect to the cheerleader effect’s theoretical explanation, suggesting that *contrast effects* cause the cheerleader effect. The attractiveness of the flanking faces is the standard of comparison for the attractiveness of the target face. Ying et al. (2019) argue that the contrast between the flanking face’s attractiveness and that of a target face boosts the perceived attractiveness in the opposite direction. This leads to the prediction that a target face is perceived as more attractive when it is flanked by less attractive faces. Thus, the relative differences between the target face and the flanking faces cause the effect. If there is no difference between the attractiveness of the target face and the flanking faces, the perceived attractiveness of the target face should not change.

### Change in the Evaluation Mode Between Separate vs. Joint Evaluations

The aim of this article is to refine this cognitive view with a change in the evaluation mode of joint and separate evaluations (Hsee and Leclerc, 1998; Hsee and Zhang, 2010). The example of purchasing a watch illustrates this change in the evaluation mode: if potential buyers are offered only a single watch, they will use internal information to build their impressions of the watch’s value. However, when buyers are offered several watches, they can compare these watches. This leads to a change in the evaluation mode. Now, the relative differences among the watches is relevant for the evaluation. The internal standard plays a crucial role. Watches below the internal standard will profit from this change in evaluation mode even when they are compared to equal or superior watches, as long as the alternatives are still below the internal standard. The opposite happens with watches above the internal standard. They do not profit when compared with other watches above the internal standard, because the relative difference between watches above the standard is less than the difference from the internal standard. Therefore, it is wise to present luxury products separately and inferior products jointly (Hsee and Leclerc, 1998). In a similar way, a change in evaluation mode could cause the cheerleader effect. The evaluation of the attractiveness of an individual face is based on internal standards. The evaluation of the attractiveness of a face in the context of flanking faces is based on comparison with the flanking faces. The attractiveness of a face increases if the accompanying are less attractive than the internal standard. This leads to the prediction that faces of low attractiveness should profit from flanking faces, even when there is no difference between the attractiveness of the target face and that of the flanking faces.

The change in evaluation mode is a further process which could cause a cheerleader effect. The contrast model and the change in evaluation mode do not contradict each other. The change in evaluation mode complements the contrast model and offers a further explanation for how a cheerleader effect could emerge. According to this theory, individual faces are evaluated in contrast to an internal standard. However, if flanking faces are available, target faces are evaluated in contrast to the flanking faces. Now two processes could cause a cheerleader effect. It is possible that the contrast between the target face and the flanking faces causes a cheerleader effect. In addition, it is possible that the change in evaluation mode, from internal standard to flanking faces, is another possible cause for this effect.

**FIGURE 1:**
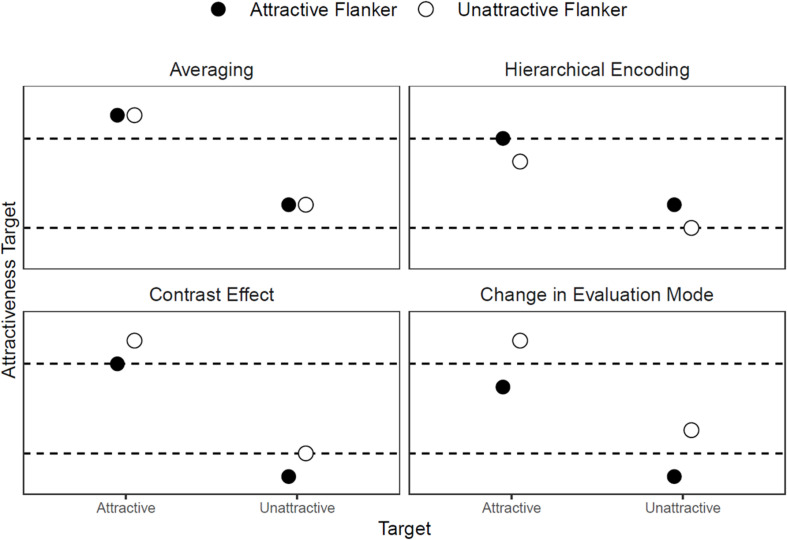
Predictions of target face attractiveness. This figure illustrates that the four theories differ in their predictions of how the attractiveness of a target face changes when it is flanked by other faces. The dashed lines represent the ratings of the attractive and unattractive faces when they are not flanked. The circles indicate the ratings of the flanked faces. Averaging visual information leads to increased perceived attractiveness, regardless of the attractiveness of the target and flankers compared to the unflanked target. According to hierarchical encoding, visual information is stored in higher-order representations. The rating of the target depends on this representation and drifts to the mean rating of all simultaneously presented faces. The *contrast effect* states that the flanking faces provide the standard of comparison for the target faces. If this standard is low, the target is rated as more attractive and vice versa. Presenting a target with flankers rather than in isolation leads to a change in the evaluation mode. The target is no longer compared to the internal standard; instead the difference between target and flankers becomes important. If there is a contrast between target and flankers, the rating becomes more extreme; if there is no contrast, the rating becomes more moderate. This figure demonstrates that the four theories differ in their predictions of how the attractiveness of a target face changes when it is flanked by other faces.

In sum, the four explanations differ in their predictions concerning the influence of the target face’s attractiveness on the cheerleader effect. Figure 1 illustrates the differences. Averaging predicts that the attractiveness of faces always increases when they are flanked by other faces. Hierarchical encoding holds that the cheerleader effect ensues when faces are flanked by more attractive faces. The contrast effect and the change in evaluation mode posit that only flankers of low attractiveness bring about the cheerleader effect; however, they differ in their predictions when there is no contrast between the attractiveness of the target face and the flanking faces. Without any difference between the attractiveness of the target face and that of the flanking faces, the contrast effect would predict no cheerleader effect; however, the change in evaluation mode predicts that less attractive target faces profit from equally less attractive flanking faces. Thus, even without any contrast between the attractiveness of the target face and the flanking faces, a cheerleader effect should occur.

Consequently, a study in which target face attractiveness and flanking faces’ attractiveness are manipulated independently would provide evidence of the processes underlying the cheerleader effect.

### Two Research Traditions

The cheerleader effect has mainly been studied by cognitive psychologists, whereas the evaluation mode has been mainly studied by behavioral scientists. As a result, the research traditions differ in their units of analysis. In cognitive psychology, faces provide the unit of analysis. A few participants rate many faces in a within-subject design and ratings for each face are aggregated. The cheerleader effect is calculated by analyzing a sample of faces. In behavioral science, the units of observation are typically participants, and often in a between-subject design. In the case of the cheerleader effect, both strategies are possible. However, they differ in the interpretation. Focusing on faces provides an impression of the variance of face attractiveness when adding flanking faces. Focusing on participants gives an impression of the variance of human judgments when adding flanking faces. Studies of the cheerleader effect have typically analyzed faces as units of observation. For this reason, we did this as well. However, we decided that each face should be rated by each participant only once. This corresponds to a situation where an observer evaluates an unfamiliar face for the first time. In this regard we differ from previous studies that have investigated the cheerleader effect.

## Materials and Methods

### Sample Size and Disclosure Statement

The sample size was determined before any data analysis was carried out. Because some studies have replicated the cheerleader effect (Carragher et al., 2018, 2019; Ying et al., 2019) while others failed to do so (van Osch et al., 2015), it is hardly possible to estimate the effect size. A recent study (Ying et al., 2019) used six faces, which were evaluated by 20 participants. In this study, the number of evaluated faces was increased to 24 and we decided to recruit 600 participants. The study was conducted with more participants than those of former studies because in this study, each participant only rated 12 different faces and each face only once. This corresponds to a situation where an observer evaluates an unfamiliar face for the first time. Thus, the attractiveness of each face in each condition was rated by at least 94 participants.

### Participants

The participants were all United States residents and were contacted via Amazon Mechanical Turk. A total of 605 participants completed the study; 19 failed to pass an attention check and were dismissed. Of the remaining 586 participants, 240 were female, 343 were male, and three identified as non-binary. The mean age was 37.16 (SD = 11.23, range = 18–73).

### Design

The study used a 3 (Flankers) × 2 (Targets’ attractiveness) mixed factorial design. Each face was rated without flanking faces, with highly attractive flanking faces, and with low attractive flanking faces. In addition, the targets’ attractiveness was manipulated: half of the participants were shown only highly attractive targets, while the other half were shown only targets of low attractiveness. Note that the highly attractive target faces did not differ in their attractiveness from the highly attractive flanker faces. Likewise, the target faces of low attractiveness did not differ in their attractiveness from the flanking faces of low attractiveness. The order in which the faces appeared was counterbalanced, as was the side on which the flankers were presented. The condition assignment was random.

A sensitivity power analysis for the mixed model ANOVA, with an alpha significance criterion of 0.05 (two-tailed), a standard power criterion of 0.8 for two groups (Targets’ attractiveness), and three repeated measures (Flankers) that highly correlate (*r* = 0.98), yielded an effect size of *F* = 0.054 (η^2^ = 0.22) for 24 faces. A sensitivity power analysis for the *t*-tests, with an alpha significance criterion of 0.05 (two-tailed) and a standard power criterion of 0.8 for matched pairs, yielded an effect size of *d* = 0.89 for 12 faces.

### Procedure and Materials

#### Dependent Variable

After participants provided their informed consent, they were instructed to rate the attractiveness of the face in the middle of the screen on a scale from 0 (*not at all attractive*) to 100 (*extremely attractive*). Each participant rated 12 faces. For each face, the mean attractiveness rating served as the dependent variable. The participants were shown faces from the Chicago Face Database (Ma et al., 2015), which contains highly standardized pictures grouped according to gender, age, and race. All faces are depicted from a frontal perspective and have a neutral facial expression. For each face, there is an attractiveness rating, which allows for a selection of faces of the same gender and race and of similar attractiveness. Twenty-four sets of faces containing three faces that did not differ in gender or race (White, Hispanic, African–American, and Asian) and that were similar in their attractiveness were selected. Half of the faces were male and half female.

#### Attention Check

Typically, online experiments contain an attention check to filter out participants who did not read the instructions carefully. After measuring the dependent variable, participants were shown a picture of a dog’s face and instructed to click on a scale from 0 to 100, but only within the range of 20–30. This served as an attention check.

#### Manipulation Check

After the attention check, participants rated the attractiveness of the flankers. To compare the flankers with the target faces with regard to attractiveness, only data from participants who had previously evaluated targets without flankers were analyzed, so that they evaluated the flankers’ facial attractiveness viewing them for the first time.

#### Further Measure

Following the manipulation check, the participants rated the attractiveness of one actor and one actress. As these data are not relevant for this article, they are not included in the results. Finally, the participants answered demographic questions.

## Results

### Manipulation Check

The manipulation of the targets’ attractiveness was successful. All the analyses were based on evaluations of faces that were presented in isolation. The targets of high attractiveness (*M* = 58.44, SD = 10.71) were more attractive than the targets of low attractiveness (*M* = 39.10, SD = 4.69), *t*(22) = 5.73, *p* < 0.001, Cohen’s *d* = 2.34.

The attractiveness of the flanking faces corresponded to the target face attractiveness: the targets of high attractiveness (*M* = 58.44, SD = 10.71) were equally attractive as the highly attractive flankers (*M* = 58.28, SD = 9.08), *t*(11) < 1, *p* = 0.94, Cohen’s *d* = 0.02. Likewise, the targets of low attractiveness (*M* = 39.1, SD = 4.69) were equally attractive as the flankers of low attractiveness (*M* = 37.81, SD = 4.13), *t*(11) < 1, *p* = 0.37, Cohen’s *d* = 0.27.

### Main Results

An ANOVA of the between-subjects factor targets (high vs. low attractiveness) and the repeated measures flankers (without flankers, low attractiveness, and high attractiveness) revealed a main effect of the targets *F*(1,22) = 42.81, *p* < 0.001, η^2^ = 0.66, a main effect of the flankers, *F*(2,44) = 99.2, *p* < 0.05, η^2^ = 0.05, and an interaction *F*(2, 44) = 20.71, *p* < 0.001, η^2^ = 0.01. Due to the manipulation, the targets of high attractiveness (*M* = 59.79, SD = 9.32) were more attractive than the targets of low attractiveness (*M* = 40.28, SD = 4.45), *t*(22) = 6.53, *p*_*Bonferroni*_ < 0.001, Cohen’s *d* = 1.33. The manipulation of the flankers resulted in a cheerleader effect. The target faces were perceived as more attractive when flanked by faces of low attractiveness (*M* = 53.77, SD = 10.51) than when they were not flanked (*M* = 48.77, SD = 12.77), *t*(23) = 8.34, *p*_*Bonferroni*_ = < 0.001, Cohen’s *d* = 1.70. In addition, target faces were perceived as more attractive when flanked by faces of low attractiveness (*M* = 53.77, SD = 10.51) than when they were flanked by faces of high attractiveness (*M* = 47.55, SD = 13.68), *t* = 7.91, *p_*Bonferroni*_* < 0.001, Cohen’s *d* = 1.61.

**FIGURE 2:**
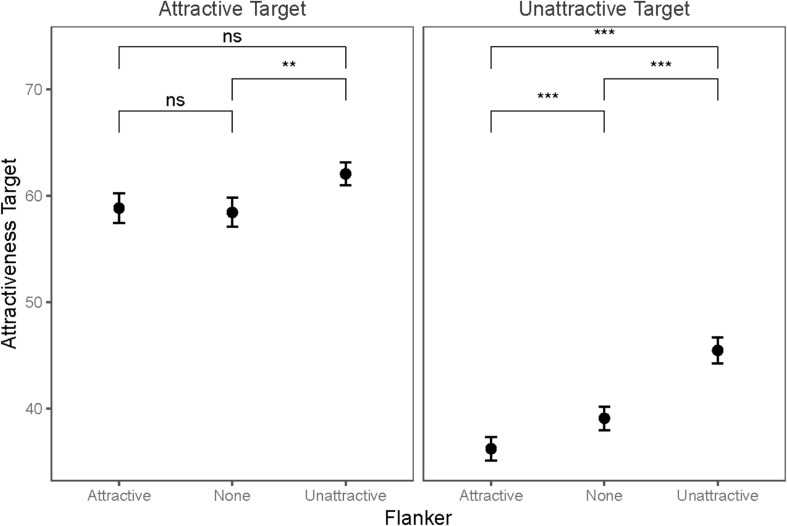
Changes in the attractiveness of faces of low and high attractiveness due to flankers of low and high attractiveness. This figure illustrates the changes in the mean and the 95% confidence interval in the attractiveness of target faces of low and high attractiveness when they are not flanked, and when they are flanked by faces of low or high attractiveness (ns = not significant; * < 0.05; ** < 0.01; *** < 0.001).

This effect was moderated by the targets’ attractiveness. The direction of the moderation was in line with the predictions of the change in evaluation mode. Figure 2 illustrates the results. The cheerleader effect emerged when there was no difference between the attractiveness of the target and that of the flanking faces. The target faces of low attractiveness (*M* = 39.10, SD = 4.69) were evaluated as more attractive when they were flanked by faces of equally low attractiveness (*M* = 45.48, SD = 4.84), *t*(22) = 9.64, *p*_*Bonferroni*_ < 0.001, Cohen’s *d* = 1.97.

In line with *contrast effects* and *change in evaluation mode* the faces of high attractiveness were in a contrast to the flankers with low attractiveness. The faces of high attractiveness without flanking faces (*M* = 58.44, SD = 10.71) were more attractive when they were flanked by faces of low attractiveness (*M* = 62.06, SD = 7.59), *t*(22) = 5.47, *p*_*Bonferroni*_ < 0.001, Cohen’s *d* = 1.12 (which is smaller than that of faces of low attractiveness, *t*(22) = 2.60, *p* = 0.20).

A reversed cheerleader effect emerged when the faces of low attractiveness (*M* = 39.10, SD = 4.69) were flanked by the faces of high attractiveness (*M* = 36.25, SD = 4.05), *t*(22) = 4.30, *p*_*Bonferroni*_ < 0.001, Cohen’s *d* = 0.88. This is in line with the predictions of the *contrast effect* and the *change in evaluation mode*. However, no changes emerged when the faces of high attractiveness (*M* = 58.44, SD = 10.71) were flanked by the faces of high attractiveness (*M* = 58.85, SD = 9.81), *t*(22) < 1.00, *p*_*Bonferroni*_ = 0.54, Cohen’s *d* = 0.13. This is in line with the predictions of the *contrast effect* and *hierarchical encoding.*

### Additional Analysis

Although there are no significant differences between the targets and flanker’s attractiveness if they are either both highly attractive or both less attractive, there are still minimal contrasts. Since we used different faces for the target and the flankers, there are always some minimal differences between the target and the flankers in terms of attractiveness. So, there may still be *contrast effects* between high attractive targets and high attractive flankers, or unattractive targets and unattractive flankers. To investigate if those minimal contrasts arouse cheerleader effects, we calculated the correlations of the contrasts between targets and flanker’s attractiveness with the cheerleader effect.

We did not find any correlation between the mean contrast between target and flankers and the cheerleader effect for attractive faces, *r*(10) = 0.04, *p* = 0.90, unattractive faces *r*(10) = 0.20, *p* = 0.54, or pooled together *r*(22) = −0.22, *p* = 0.30.

However, we found correlations between the contrast and the cheerleader effect for attractive faces flanked by unattractive faces *r*(10) = 0.86, *p* < 0.001 and for unattractive faces flanked by attractive faces *r*(10) = 0.66, *p* < 0.05.

## Discussion

The goal of this study was to demonstrate that a change in evaluation mode could cause a cheerleader effect. The results show that faces are perceived as more attractive when they are flanked by faces of low rather than high attractiveness, even when the target faces do not differ in attractiveness from the flanking faces. This is in line with the predictions of the change in evaluation mode and that the presence of flanking faces changes the evaluation mode (Hsee and Leclerc, 1998; Hsee and Zhang, 2010).

### Contrast Effect and Evaluation Mode

The contrast hypothesis and the evaluation mode do not contradict each other. Both theories argue that judgments are constructed by contrasts. When flanking faces are available, target face attractiveness is evaluated in contrast to flanking faces. The contrast between the target face and the flanking face could cause a cheerleader effect (Ying et al., 2019). However, if no flanking faces are available, observers base their judgment on the contrast with their internal standards (Hsee and Zhang, 2010). This change from an external to an internal standard of comparison could cause a cheerleader effect as well. In our experiment, we minimized the contrast between the target faces and the flanking faces. In the condition with unattractive targets flanked by equally unattractive flankers, we observed a cheerleader effect.

In our experiment, we had no direct measure of the change in evaluation mode. Our argumentation is based on the idea that a contrast between target and flanking face attractiveness is a necessary condition for a contrast effect. Therefore, we selected targets and flankers which are very similar in their degree of attractiveness. However, minimal contrasts between target and flanking face attractiveness still exist. Therefore, we cannot rule out the possibility that minimal contrasts cause the cheerleader effect in those conditions as well. However, there are additional results which support our hypotheses. First, when considering faces with low attractiveness with equally low attractive flanking faces the cheerleader effect is greater than when considering highly attractive faces with unattractive flanking faces, although the contrasts are smaller. Second, we calculated the difference between the attractiveness of each target face with equally attractive flankers (minimal contrasts) and with more or less attractive flankers respectively (high contrast). In both conditions with high contrast there was a correlation between the contrast and the cheerleader effect. However, in both conditions with minimal contrast there was no correlation between the contrast and the cheerleader effect.

The aim of this paper is to introduce the idea that a change of evaluation mode is a process which could cause a cheerleader effect. Falsifying other processes is not an aim of this paper. Actually, even small changes could cause other processes to influence the evaluation of facial attractiveness.

### Real-Time Rating vs. Memory

This study focused on real-time impressions and not on memory-based judgments. Therefore, the participants rated the attractiveness of faces online while these faces were in view. However, real-time ratings differ from memory-based judgments (Hastie and Park, 1986; Ying et al., 2020). It is possible that visual memory processes have a higher influence on attractiveness ratings when judgments are memory-based but not when they occur in real time. In a recent study, participants evaluated faces after they had disappeared from the screen (Ying et al., 2019). Although the interval was short, the participants gave memory-based judgments. Ying et al.’s results could be interpreted as a mix between cognitive and visual memory processes because they show that facial attractiveness was more favorable when faces were flanked by faces of both low and high attractiveness.

### Simultaneous and Sequential Presentation of Faces

Similar to memory-based judgments are situations where people evaluate a face online and compare it with a formerly viewed face. Such situations attest to two opposing influences: on the one hand a face is rated as more attractive when it follows a face of low attractiveness (Pegors et al., 2015; Ying et al., 2019), while on the other hand a face is rated as more attractive when a face of high attractiveness precedes it (Pegors et al., 2015). Therefore, judgments of the perceived attractiveness of flanked faces may differ when they are recalled compared to when they are made in real time. In addition, there is evidence that the cognitive processes differ if the observer evaluates a group of faces simultaneously or sequentially (Ying et al., 2020).

### First Impressions vs. Familiar Faces

One important limitation of our study concerns the familiarity of the faces. Similar to other studies of the cheerleader effect, we measured the attractiveness of faces that were unfamiliar to the participants. Therefore, our results are based exclusively on the first impression of these faces. The precise mechanisms by which the attractiveness of a familiar face is influenced by flanked faces remains to be determined. However, attractiveness judgments are not only influenced by physical aspects but also by psychological aspects, such as associations (Rhodes and Zebrowitz, 2002) or sentimental feelings (Yang and Galak, 2015). It is possible that the more an attractiveness rating is influenced by psychological aspects, the less it is influenced by flanking faces.

### Highly Attractive Flankers

It seems that a reversal of the cheerleader effect is less likely to occur than the cheerleader effect. In the present study, we found a reversal of the cheerleader effect when target faces were flanked by highly attractive faces only for target faces of low attractiveness, but not when highly attractive target faces were flanked by equally highly attractive faces. Similarly, Ying et al. (2019) reported cheerleader effects and no reversal of the cheerleader effect even when the flankers were attractive. One possible explanation is that in addition to cognitive processes, additional processes, such as averaging in the visual memory, generally increase facial attractiveness in groups.

### Extremely Attractive Faces

A further limitation pertains to extremely attractive faces. We did not use extremely attractive faces. The potential to increase the attraction of extremely attractive faces is limited. Therefore, due to the ceiling effect, one would expect no or minimal cheerleader effects for extremely attractive people. In addition, if a person is unambiguously attractive, like Scarlett Johansson or Chris Hemsworth, observers do not need additional information to build their impressions. They have sufficient information for their evaluation, will not contrast them to flanking faces, and will not sample additional information (Simon, 1955; Fiedler and Bless, 2010; Stüttgen et al., 2012). However, for people with more ambiguous levels of attractiveness, such as John C. Reilly or Rebel Wilson, observers will consider the attractiveness of flanking faces (Messner, manuscript in preparation).

### Assimilation vs. Contrast

Judgments are not always formed in contrast to something; they can be formed in assimilation toward something as well (Sherif et al., 1958; Mussweiler, 2003; Bless and Schwarz, 2010). Assimilation corresponds to the idea of hierarchical encoding. An explanation of the cheerleader effect based on hierarchical encoding is based on two assumptions: First, observers calculate the mean attractiveness of faces they see simultaneously; second, observers differentiate between the target face and other faces and bias their evaluation of the attractiveness of the target face toward the main attractiveness of the group of other faces. While evidence for the first assumption exists (Luo and Zhou, 2018), no such evidence exists for the second assumption (Luo and Zhou, 2018; Carragher et al., 2019; Ying et al., 2019). However, it is possible that additional redundancy would facilitate differentiation between the target face and the flanking faces and foster hierarchical encoding.

## Conclusion

The change in evaluation mode has a high impact on marketing practice. A seller of low-budget products (e.g., a cheap-looking watch) presents the products alongside other low-budget products (other cheap-looking watches), while the seller of luxury goods presents the products separately (Hsee and Leclerc, 1998). This article provides evidence that similar processes are relevant for self-marketing, assuming the goal is that observers evaluate one’s attractiveness highly when one posts selfies on social media. One appears more attractive in a selfie with other people than in isolation, as long as the other people are equally or less attractive. The higher one’s own attractiveness, the less one benefits from this effect; however, it is not beneficial to post a selfie taken with other people in the frame if the attractiveness of these other people is high. Finally, the more unambiguous one’s attractiveness, the less one is affected by flanking faces.

## Data Availability Statement

The datasets presented in this study can be found in online repositories. The names of the repository/repositories and accession number(s) can be found below: the material and the data are available at https://osf.io/qhjxs/?view_only=0d857d0514604aeca912a3202ee3c74f.

## Ethics Statement

The studies involving human participants were reviewed and approved by Ethics Committee of the Faculty of Business, Economics, and Social Science of the University of Bern (Project Number: 102019). The patients/participants provided their written informed consent to participate in this study.

## Author Contributions

All authors contributed to the study design, analyzed the data and approved the final manuscript. CM wrote the manuscript. MC and PH contributed their input.

## Conflict of Interest

The authors declare that the research was conducted in the absence of any commercial or financial relationships that could be construed as a potential conflict of interest.
